# Xantogranulomatous Salpingo Oophritis, Lessons Learnt: Report of Two Cases With Unusual Presentation 

**Published:** 2017-09

**Authors:** Ruchi Rathore, Shivangi Chauhan, Suman Mendiratta, Ritu Sharma, Manupriya Nain, Namrata Sarin

**Affiliations:** 1 Department of Pathology, NDMC and Hindu Rao Hospital, Delhi, India; 2 Department of Obstetrics and Gynaecology, NDMC and Hindu Rao Hospital, Delhi, India

**Keywords:** Xanthogranulomatous Inflammation, Ovary, Fallopian Tube, Tuberculosis, Malignancy

## Abstract

Xanthogranulomatous inflammation is a rare form of chronic inflammatory response consisting of macrophages, lymphocytes, plasma cells and neutrophils too. Due to its locally destructive nature and mass forming capacity as a result of adhesions, this type of inflammation may mimic malignancy or tuberculosis both clinically and radiologically. We present a report of two such cases, one mimicking tuberculosis and the other mimicking malignancy clinically. Awareness of this condition and a higher index of suspicion among clinicians, radiologists and pathologists can help in early diagnosis and more appropriate treatment of this potentially destructive disorder.

## Introduction

Xanthogranulomatous inflammation is a well known histopathological entity. More commonly described in the gall bladder and kidney, this form of inflammation is uncommon in the fallopian tubes and ovaries. It is a relatively rare form of chronic inflammatory response that causes destruction of the affected organs. Dominated by the presence of lipid containing foamy histiocytes, there are sheets of mixed inflammatory infiltrates consisting of lymphocytes, plasma cells, neutrophils and multinucleated giant cells in the tissue parenchyma causing destruction (1). However due to its locally destructive nature and mass forming capacity as a result of adhesions, this type of inflammation may sometimes mimick malignancy both clinically and radiologically. Since it effects a vast majority of organs apart from gall bladder and kidney including urinary bladder, testis, stomach and female genital tract, awareness of this entity is of paramount importance in proper management of such patients (2). We present a report of two cases with xanthogranulomatous inflammation, unique in terms of their presentation one mimicking tuberculosis and other misdiagnosed as malignancy clinico-radiologically.

## Case Report 1

A 21 year old female presented to gynecology out patient department with complaints of inability to concieve for the last 5 years. Her menstrual history was within normal limits except for the last 5 months whe she developed irregular menstrual bleeding and dysmennorhea. There was no history of any chronic illness like tuberculosis in the past. She had undergone appendicectomy 11 years back for acute appendicitis. On general physical examination she had pallor and mild pedal edema. On per abdomen examination there was a lump in abdomen measuring approximately 8 X 8 centimeters in the left iliac fossa. Her per vaginum examination revealed bilateral tender fornices and a bulky uterus while per speculum examination showed healthy vagina and cervix. 

Patient was further investigated and on Ultrasonography was found to have left sided ovarian cyst measuring 5 X 4 cm, diagnosed as a complex cyst with septations. Uterus was found to be anteverted, bulky and with multiple fibroids both on anterior and posterior walls. Kidney also showed hydronephrotic changes. On Hysterosalpingography, bilateral tubes were blocked. Laparotomy was perfomed which revealed a large tubo-ovarian mass on left side measuring 6 X 7 cm. Omentum and bowel were found to be adherent to this mass. Along with a large 5 X 2 cm fibroid found in anterior wall of  uterus. Right sided falllopian tube was tortuous and edematous while right ovary was apparently normal. So a Left salpingo ophrectomy was performed and sent for histopathological examination. Pus drained from omentum was sent for culture and sensitivity. However it did not reveal any growth (including Mycobacterium tuberculosis) even after 4 weeks .Other investigation of the patient revealed CA 125 levels to be 246 U/ml and serum LH levels to be 7.58mIU/ml. Thyroid profile was normal.

**Figure 1:**
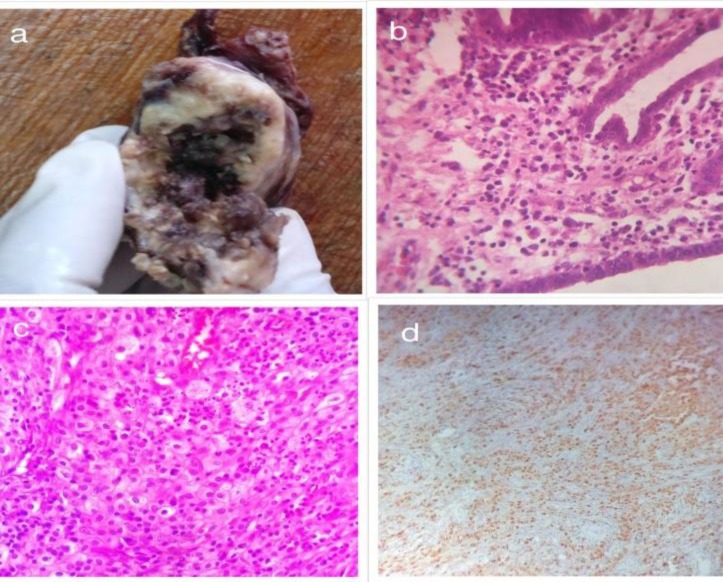
(A) Gross specimen of case 2 showing yellowish areas which revealed xanthomatous inflammation on microscopy. (B) 100x of fallopian tube of case 2 showing tubal lining and xanthomatous inflammation. (C) 400x of ovary of case 1 showing foamy macrophages, lymphocytes and neutrophils. (D) Immunohistochemistry for CD68 showing positivity in more than 60 percent cells.

***Histopathological examination: ***We received a mutilated specimen in three pieces. The largest piece measured 5×3×2 cm. Outer surface was exudate covered and congested. Cut surface revealed red brown areas with a luminal structure likely to be tube. Second and third pieces were measuring 4×3×1 cm and 1×1×0.5 cm. Sections from the ovary showed sheets of foamy histiocytes along with the presence of inflammatory infiltrates in the form of lymphocytes, plasma cells and some neutrophils and eosinophils. There were fair number of foamy histiocytes with abundant lipid laden vacoules in the cytoplasm and hypochromatic nuclei. There was fibrosis along with some vascular proliferation in the ovarian parenchyma. Sections from fallopian tubes also showed the presence of xanthogranulomatous inflammation in the lamina propria and the serosa of the tube walls (as shown in figure 1). Periodic Acid Schiff (PAS) and Acid fast stains were negative. Subsequent immunohistochemical stains demostrated positive CD68, CD 3 snd CD 20 were suggestive of a mixed inlflammatory infilterates in both tube and ovary. Based on the above features a diagnosis of xanthogranulomatous salpingo ophritis was rendered. 

## Case Report 2

A 43 year old women presented to gynaecological out patient department with complain of lump in abdomen. On per abdomen examination her abdomen was soft with a left sided lump measuring 4 × 4 cm. On per speculum examination cervix was found to be hypertrophied and on per vaginum she was found to have a bulky uterus with left sided mass measuring approximately 6×6 cm. Bilateral fornices were free. Patient had a history of cholecystectomy 11 years back.

Her ultrasound examination revealed a left sided anechoic ovarian mass measuring 6.4 × 5.3 × 4.5 cm. Her magnetic resonance imaging also showed a well defined, thick walled, irregular, left ovarian cystic lesion with peri ovarian and parametrial fat straddling. On suspicion of a neoplastic condition she was planned for a total abdominal hysterectomy with bilateral salpingo oophorectomy. On laparotomy she was found to have a 6 × 6 cm solid cystic mass in left para ovarian region. Left fallopian tube and sigmoid colon were also adherent to the mass. A part of tissue was sent for frozen section which revealed fibroconnective tissue with presence of inflammatory infiltrate in the form of neutrophils, plasma cells, lymphocyte and histiocytes only. No tumor was identified. Culture from peritoneal fluid were negative for mycobacterium tuberculosis. 

***Histopathological examination: ***Total abdominal hysterectomy with bilateral salpingo oophorectomy was performed and sent for histopathological examination with a clinical diagnosis of ovarian neoplasm. Uterus measured 8 × 11 × 4 cm with attached right tube measuring 4 × 1 cm and ovary measuring 3×1 cm. Cut sections revealed multiple cysts filled with mucoid material measuring 0.5 to 1 cm. The left sided adnexal mass measured 6 × 5 × 2 cm with solid cystic cut section filled with grey brown mucoid material at some foci.

Microscopic examination of complete specimen showed areas with xanthogranulomatous inflammation consisting of foamy histiocytes, lymphocytes, plasma cells and neutrophils infiltrating the ovarian stroma and fallopian tubes (as shown in figure 1) suggestive of xanthogranulomatous salpingo oophoritis. Immunohistochemistry for CD 68 showed positivity confirming the presence of histiocytes.

## Discussion

Xanthogranulomatous inflammation of uterus, fallopian tube and ovary was first described by Kunakemakorn in 1976 (1). To date, only very few cases of xanthogranulomatous salpingo oophoritis have been reported. Although the exact cause of this uncommon form of chronic inflammatory response is still unknown, the presence of endometriosis, pelvic inflammatory disease, intrauterine contraceptive device, an ineffective antibiotic therapy or an abnormal lipid metabolism are considered to be the main causes of this disease (2) (3). Infections by various organisms like Escherichia coli, Proteus vulgaris, bacteroides fragilis and salmonella typhi have been associated with such inflammation. However, in both our cases history of PID, IUD insertion could not be elicited. Some authors also argue that the since it is a chronic inflammatory process leading to tissue necrosis, the continuous release of cholesterol and other lipids from the dead cells, phagocytosed by macrophages, leads to a xanthomatous process (4). In our first case the patient was thought to have tuberculosis on laparotomy due to extensive tissue destruction of the surrounding organs but the cultures of the same were not found to be positive for Mycobacterium tuberculosis. Moreover the pus culture sensitivity for other anaerobic bacterias attributed to the disease was also negative. In our second case the clinical and radiological findings were suspicious for tumor owing to the mass forming nature and involvement of surrounding fat, however, the diagnosis of malignancy could be rebuked on frozen section itself and confirmed on further histopathological sections because of the awareness of this condition by the pathologist. 

The average age for this lesion is 38.5 years (range 23-72) and the youngest case reported was of 18 years (5). Our first patient being 21 years old was relatively young for this presentation.

Clinically these patients usually present with fever, abdominal mass, menorrhagia, pain in abdomen or anemia (6). The presentation with primary infertility with no prior history of menorrhagia or endometriosis is also one of the key features in our first case. Accordingly as in our patient, the erythrocyte sedimentation rate and total leukocyte count are found to be raised in these patients. More often these lesions tend to form tumor like masses as in our second case due to the fibrosis and adhesions resulting form chronic inflammation. The importance of recognizing these locally destructive mass forming lesions comes from the fact that these may be misdiagnosed both clinically and radiologically as a malignant mass forming lesion. Also in a country like ours where the incidence of tuberculosis is high, these lesions could also mimic tuberculosis and be started on Antitubercular treatment as happened in our first case. 

Grossly the affected ovary is usually enlarged, varying in size from 3-17 cm (7-15). In our first case the salpingoophrectomy specimen was mutilated and received in 3 pieces approximately measuring 5 cm. According to the literature ovary is not only replaced by tumor like solid yellow, nodular mass that may be cystic due to liquefactive necrosis but it also adheres to the adjacent organs and the pelvic peritoneum leading their destruction further arousing a suspicion of malignancy as in our second case (14). 

Microscopically these lesion are characterized predominantly by lipid laden macrophages along with dense inflammatory infiltrates of lymphocytes plasma cells and polymorphonuclear leucocytes. In our case while the ovary was completely involved in the process with little preserved parenchyma ,the fallopian tube showed this inflammation predominantly in the lamina propria in both the cases suggesting a contiguous involvement of fallopian tube and ovary. 

Both non-neoplastic and neoplastic conditions can be included in the differential diagnosis. The non neoplastic conditions primarily include infections like tuberculosis and fungal infections which as in our case were ruled out by performing special stains (AFB and PAS). One of the most important differentials resembling xanthogranulomatous inflammation microscopically is malakoplakia where concentric calcific bodies (Michaelis- Gutmann bodies) are found in the cytoplasm, which were not seen in either of our cases. 

Sometimes neoplastic conditions like lymphoma or leukemia, malignant small cell tumor and sclerosing stromal tumor may also be considered in the list of differential diagnosis. However since we had a polymorphous population of inflammatory cells confirmed on immunohistochemistry by CD 68, CD3 and CD 20, we ruled out these possibilities (4).Thus clinically, radiologically and sometimes pathologically xanthogranulomatous oophoritis can be confused with ovarian malignancy (2).

As done in our cases, surgery is the treatment of choice. But an Awareness of this inflammatory lesion among the clinicians, radiologists and pathologists may not only prevent overdiagnosis and extensive surgeries for the patients but may also reduce morbidity giving a better prognosis to these patients (16).

## Conclusion

Although the exact etiology of this locally desructive inflammatory lesion is unknown, we suggest that patients with PID, endometriosis, and IUCD etc. should be kept under follow up to prevent the occurrence of this disease. Awareness of this condition and a higher index of suspicion among clinicians, radiologists and pathologists can further help in early diagnosis and more appropriate treatment of this potentially destructive disorder.
